# Plasma Glial Fibrillary Acidic Protein (GFAP) shows age-dependent associations with externalizing psychopathology and atypical brain connectivity

**DOI:** 10.1038/s41398-026-04114-2

**Published:** 2026-05-29

**Authors:** B. S. Niveditha, Bharath Holla, Sarada Subramanian, N. Gagana, K. M. Bhargavi, Eesha Sharma, Jayant Mahadevan, Meera Purushottam, Biju Viswanath, Vivek Benegal, Gautham Arunachal, Jon Heron, Matthew Hickman, Debasish Basu, B. N. Subodh, Lenin Singh, Roshan Singh, Kalyanaraman Kumaran, Rebecca Kuriyan, Sunita Simon Kurpad, Kamakshi Kartik, Kartik Kalyanram, Sylvane Desrivieres, Gareth Barker, Dimitri Papadopoulos Orfanos, Mireille B. Toledano, Pratima Murthy, Nilakshi Vaidya, Ghattu Krishnaveni, Gunter Schumann, Kuldeep Kumar Sharma, Binukumar Bhaskarapillai, K. Thennarasu, Rajan Kashyap, Rose Dawn Bharath, Amit Chakrabarti, G. K. Chetan, Muchukunte Mukunda Srinivas Bharath

**Affiliations:** 1 Department of Human Genetics, National Institute of Mental Health and Neurosciences (NIMHANS), No. 2900, Hosur Road, Bengaluru, 560029 Karnataka India; 2 Department of Clinical Psychopharmacology and Neurotoxicology, NIMHANS, Bengaluru, Karnataka India; 3 Department of Integrative Medicine, NIMHANS, Bengaluru, Karnataka India; 4 Department of Neurochemistry, NIMHANS, Bengaluru, Karnataka India; 5 Department of Child and Adolescent Psychiatry, NIMHANS, Bengaluru, Karnataka India; 6 Department of Psychiatry, NIMHANS, Bengaluru, Karnataka India; 7 Rohini Nilekani Centre for Brain and Mind, Department of Psychiatry, NIMHANS, Bengaluru, Karnataka India; 8 Population Health Sciences, Bristol Medical School, University of Bristol, Beacon House, Queens Road, Bristol, BS8 1QU United Kingdom; 9 Department of Psychiatry, Post Graduate Institute of Medical Education and Research, Chandigarh, 160012 India; 10 Department of Psychiatry, Regional Institute of Medical Sciences, Lamphel Road, Lamphelpat, Imphal, Manipur 795004 India; 11 Department of Psychology, Regional Institute of Medical Sciences, Imphal, 795004 India; 12 Primary Care, Population Sciences and Medical Education, University of Southampton, Southampton, United Kingdom; 13 Epidemiology Research Unit, CSI Holdsworth Memorial Hospital, Mysuru, 570001 Karnataka India; 14 Division of Nutrition, St John’s Research Institute, Bengaluru, 560034 India; 15 Department of Psychiatry, St. John’s Medical College and Hospital, Bengaluru, 560034 Karnataka India; 16 Department of Medical Ethics, St. John’s Medical College and Hospital, Bengaluru, 560034 Karnataka India; 17 Rishi Valley Rural Health Centre, Madanapalle, Chittoor, Andhra Pradesh 517352 India; 18 Centre for Population Neuroscience and Precision Medicine, Institute of Psychology, Psychiatry & Neuroscience, MRC SGDP Centre, King’s College London, 16 De Crespgny Park, SE5 8AF London, United Kingdom; 19 Department of Neuroimaging, Institute of Psychology, Psychiatry and Neuroscience, King’s College London, London, SE5 8AF United Kingdom; 20 NeuroSpin, CEA, Université Paris-Saclay, Paris, France; 21 Mohn Centre for Children’s Health and Wellbeing, School of Public Health, Imperial College London, London, United Kingdom; 22 Centre for Population Neuroscience and Precision Medicine, Charite Mental Health, Dept. of Psychiatry and Psychotherapy, Charite Universitaetsmedizin Berlin, Berlin, Germany; 23 Centre for Population Neuroscience and Precision Medicine, Institute for Science and Technology of Brain-Inspired Intelligence, Fudan University, Shanghai, China; 24 Department of Biostatistics, NIMHANS, Bengaluru, Karnataka India; 25 Department of Neuroimaging and Interventional Radiology, NIMHANS, Bengaluru, Karnataka India; 26 ICMR-Centre for Ageing and Mental Health, Indian Council of Medical Research, Block-DP1, Sector-V, Salt Lake, Kolkata, 700 091 India; 27 Centre for Neurobehavioral Toxicology, Department of Clinical Psychopharmacology and Neurotoxicology, NIMHANS, Bengaluru, Karnataka India

**Keywords:** Biomarkers, Neuroscience

## Abstract

Externalizing disorders are common neurodevelopmental conditions, yet their underlying biology is not fully understood. Integrating peripheral biomarkers with brain imaging offers a powerful approach to elucidate the pathophysiology of these disorders. This study aimed to investigate the association between indicators of glial activation (glial fibrillary acidic protein; GFAP) and axonal injury (neurofilament light chain; NfL) in plasma with functional brain connectivity, and externalizing psychopathology (EXT) in a neurodevelopmental cohort. Towards this, a cross-sectional study was conducted with 144 participants selected from the Indian cVEDA cohort and balanced into EXT and healthy control (HC) groups using Mahalanobis distance matching. Plasma GFAP and NfL were quantified using Simoa technology. Resting-state fMRI data were used to generate between-network connectivity deviation scores via normative modelling. We used Gamma General Linear Models (Gamma GLMs) to test for an age-by-EXT interaction on GFAP/ NfL levels and sparse partial least squares (sPLS) regression to identify connectivity features that correlated with them. We found a significant age-by-EXT interaction for GFAP (p = 0.002), where EXT was associated with higher GFAP levels only in younger participants (<14 years). No significant effects were found for NfL. The sPLS analysis identified a significant five-feature brain connectivity signature that correlated with GFAP levels. This pattern was characterized by atypically strong connectivity between sensorimotor-limbic and attention-default mode networks, and weaker-than-expected connectivity within the default mode network. In conclusion, our findings identify a strong association between plasma GFAP and EXT in youth in an age dependent manner, suggesting a key role for glial activation in the early pathophysiology of these disorders. This process is linked to a specific, multivariate pattern of brain dysconnectivity, providing a potential neurobiological signature that warrants further investigation.

## Introduction

Externalizing psychopathology (EXT) is seen across neurodevelopmental and disruptive behaviour disorders, which are among the most prevalent and impairing conditions in adolescents and youth. These disorders are characterized by core features such as impulsivity, disinhibition, and aggression, and are strongly associated with an increased risk for later-life problems such as substance use disorders, mood disorders and other non-communicable disorders underscoring their significance as a major public health concern [1, 2]. While the behavioural manifestations of EXT are well-documented, the neurobiological mechanisms underlying these conditions remain only partially understood, which has limited the development of targeted and effective interventions [3]. There is a pressing need for objective biomarkers that can clarify the pathophysiology and track the developmental trajectories of these disorders [4].

Recent advances in molecular psychiatry have highlighted the promise of blood-based biomarkers as accessible indicators of central nervous system (CNS) processes. In particular, glial fibrillary acidic protein (GFAP) and neurofilament light chain (NfL) have emerged as sensitive markers of astrocyte activation and neuroaxonal injury, respectively [5, 6]. Quantifying these proteins in peripheral blood offers a valuable window into the neuroinflammatory and neurodegenerative processes that may contribute to psychopathology [7, 8].

GFAP, a cytoskeletal protein in astrocytes, is a widely used marker of reactive astrogliosis. Although its elevation is typically linked to major CNS injuries and neuronal death [6, 9], GFAP is also a sensitive indicator of more subtle neuropathological changes, such as synaptic loss, demyelination, and altered neural plasticity, even in the absence of overt neuronal loss [9–11]. Importantly, astrocytes are critical for brain maturation, and GFAP levels can reflect not only pathological changes but also adaptive plasticity in response to physiological stimuli such as physical activity, environmental enrichment, hormonal fluctuations, and circadian rhythm alterations [12–14].

However, the understanding of GFAP’s normative trajectory is limited. While an age-dependent downregulation in the fetal brain is reported [15], its profile across childhood and adolescence remains poorly characterized. Similarly, though GFAP levels have been examined in externalizing [16] and other neurodevelopmental disorders [17], the age-dependent dynamics within these conditions have not been systematically assessed. Therefore, quantifying plasma GFAP across the developmental course of externalizing disorders presents a unique opportunity to investigate underlying glial-related mechanisms.

On the other hand, NfL serves as a robust indicator of neuroaxonal integrity [18]. NfL is a structural component of the neuronal cytoskeleton, predominantly found in large-caliber myelinated axons, and is released into the extracellular space and subsequently into the bloodstream following axonal injury or degeneration. Elevated NfL levels in blood are considered a sensitive and dynamic marker of neuroaxonal damage, reflecting both acute and chronic injury across a range of neurological and psychiatric conditions [5]. Although not disease-specific, increased NfL concentrations reliably indicate the presence of neuroaxonal pathology, making it a valuable biomarker for monitoring neuronal health and integrity [18]. Plasma levels of GFAP and NfL are considered to be robust indicators of their brain levels [10, 19]. Together, the measurement of GFAP and NfL in plasma provides a minimally invasive approach to probing both glial and axonal processes, offering new opportunities to elucidate the neurobiological underpinnings of externalizing disorders.

To understand how these cellular-level processes manifest at the level of brain circuitry, neuroimaging techniques, especially resting-state functional MRI (rsfMRI), are useful for non-invasive mapping of large-scale brain networks. The application of normative modeling to rsfMRI data offers a powerful approach for quantifying individual deviations from typical developmental trajectories, providing a sensitive, person-specific measure of atypical brain function [20, 21]. This approach is particularly valuable for capturing the heterogeneity of brain connectivity patterns associated with externalizing disorders [4].

Despite the distinct advantages of these parallel approaches, research that integrates blood-based biomarkers and neuroimaging measures in externalizing disorders remains limited. To address these gaps, the present study leveraged data from the Consortium on Vulnerability to Externalizing Disorders and Addictions (cVEDA), focusing on a well-characterized children, adolescents and young adults sample from India [22, 23]. We specifically sought to: 1) determine if plasma GFAP and NfL levels were associated with EXT, and whether this relationship was moderated by age; and 2) identify a multivariate signature of brain connectivity deviations that associates the plasma levels of these proteins. We hypothesized that externalizing disorders would be linked to evidence of glial activation, particularly in younger individuals, and that this would be associated with a distinct pattern of atypical functional brain connectivity.

## Materials and methods

### Study design

The current study based on a cross-sectional design leveraged on a previously established large neurodevelopmental cohort (cVEDA) of children, adolescents and young adults (aged 6-23 years) from the community and from psychiatric clinics, across seven sites in India. The neuroimaging and neurocognitive data were analysed along with protein analysis on the linked biorepository plasma samples.

### Ethics statement

In the cVEDA study, written informed consent was obtained from all adult participants, and parental consent along with child assent was obtained for participants under 18 years of age. The present work involves secondary analyses of the existing dataset and analyses of plasma samples from the biobank using de-identified cVEDA data, with no additional contact with participants, following approval from the cVEDA project executive committee and NIMHANS ethics committee [Approval no. (i) Item No. VII, Sl. no. 7.08, behavioural sciences and (ii) NIMHANS/IEC (BS &NS DIV.)/2022 dated April 20, 2022]. All the methods were performed in accordance with the guidelines and regulations of the NIMHANS ethics committee and cVEDA project executive committee.

### Participants

The overall cVEDA cohort includes data on ~9000 participants recruited across different sites in India [22, 24]. Of these, 144 participants from the NIMHANS site were selected based on the availability of stored plasma samples and deep phenotyping data including structural and functional MRI. To reduce confounding in this observational design, Mahalanobis distance matching was performed using the MatchIt package in R, resulting in a 2:1 matched design of healthy controls (HC) to individuals with externalizing psychopathology (EXT). Matching was done on age, sex, Body Mass Index (BMI), general cognition factor score (gFc), and frequency of adverse childhood experiences. These variables were selected based on prior literature linking them to brain connectivity patterns, and the development of EXT [25–27]. Table 1 summarizes the demographic and clinical characteristics of the matched samples.

**Table 1:** Sample characteristics.

	Externalizing Group (n = 47)	Control Group (n = 90)	p value
Age (years)	15.63 (4.21)	15.96 (4.55)	0.686
Gender (M)	40 (85.1%)	64 (71.1%)	0.069
Body Mass Index (kg/m^2^)	18.34 (3.76)	18.75 (4.48)	0.597
General Factor Cognition (T-score)	49.52 (10.57)	50.25 (9.74)	0.684
Adverse Childhood Experiences (Frequency Score)	2.43 (1.83)	2.12 (1.73)	0.342
Run (B2) (Batch control)	22 (46.8%)	47 (52.2%)	0.547
SDQ Conduct Problems	4.99 (2.07)	2.62 (1.77)	<0.001
SDQ Hyperactivity	5.47 (2.14)	3.40 (2.05)	<0.001
SDQ Emotional Problems	3.46 (2.83)	2.74 (2.12)	0.101
SDQ Peer Problems	3.17 (2.15)	1.78 (1.81)	<0.001
SDQ Prosocial Behaviours	7.96 (2.30)	9.13 (1.57)	<0.001
**Diagnosis**			
ADHD	25 (53.2%)	–	–
ADHD and SUD	3 (6.4%)	–	–
ADHD and CD	9 (19.1%)	–	–
CD	3 (6.4%)	–	–
CD and SUD	1 (2.1%)	–	–
SUD	3 (6.4%)	–	–
ASPD	2 (4.3%)	–	–
ASPD and SUD	1 (2.1%)	–	–

*SDQ* Strength and Difficulty Questionnaire, *ODD* Oppositional Defiant Disorder, *ADHD* Attention Deficit and Hyperactivity Disorder, *CD* Conduct Disorder, *ASPD* Anti-social Personality Disorder, *SUD* Substance Use Disorder.

EXT group classification was based on structured diagnostic interviews as earlier described [24]. For participants under 18 years, psychiatric diagnoses in the EXT group were established using the Mini-International Neuropsychiatric Interview for Kids (MINI-KID) v5 and the adult MINI v5, supplemented by the Adult ADHD Self-Report Scale (ASRS v1.1) for adults (≥18 years). Participants were considered screen-positive for Adult ADHD if they checked four or more shaded responses on items 1–6 (Part A), consistent with ASRS guidelines. This threshold yields a sensitivity of 68.7% and specificity of 99.5% for ADHD, as reported earlier [28]. HC were defined by the absence of any current or past major psychiatric diagnosis on the MINI. Childhood adversity was assessed using the WHO Adverse Childhood Experiences- International Questionnaire (ACE-IQ). A gFc was derived via second-order confirmatory factor analysis from nine neuropsychological tasks included in the cVEDA battery [22].

### Sample size justification

While modest, the sample size is sufficient for detecting medium-to-large effects using bootstrapped estimation and sparse multivariate modeling. A post hoc power analysis based on the observed effect size for EXT in the Gamma General Linear Models (Gamma GLMs), indicated >90% power (n = 137, α = 0.05, two-tailed), supporting the adequacy of the sample for the primary aims of the study, and its conditional pattern was investigated using a Johnson–Neyman (JN) analysis.

### Quantification of plasma GFAP and NfL by single molecular array (SIMOA)

Blood plasma samples collected at the same time point as MRI acquisition during the baseline visit and stored at -80 ^0^C in the NIMHANS biorepository as part of the original project were processed subsequently for GFAP/ NfL estimation. SIMOA assays were performed on pseudonymized cVEDA IDs, with laboratory staff blinded to all clinical data and group labels. GFAP and NfL were measured in plasma (n = 144) in two batches using SIMOA technology on a HD-X Analyser (Quanterix, USA). The SIMOA Neurology 2-plex B kit (Quanterix, Cat. no. 103520) was utilised as per the manufacturer’s instructions. Briefly, 55 µl of each plasma sample was individually mixed with 165 µl of diluent and 200 µl of this mix was loaded onto the analyser. The preparation of calibrators and controls was carried out as indicated in the kit. The assay’s lower limit of quantification was 4.15 pg/ml, and the lower limit of detection was 0.475 pg/ml. The direct output data from the analyser were used for all subsequent analyses. The intra- and inter-assay coefficients of variation (CVs) were <10% and <15%, respectively. Among the 144 samples, only 137 provided usable GFAP/ NfL data; the same 7 samples did not yield any defined value for both NfL and GFAP. Technical analysis revealed that the values were outside the calibration range i.e. <0.2 pg/ml and >500 pg/ml for NfL; <4 pg/ml and >10 ng/ml for GFAP and hence were not considered.

### Moderation analysis

Moderation analyses were performed to test whether the relationship between EXT and GFAP/ NfL was dependent on age. Using separate Gamma GLMs, the Age-by-EXT interaction on both GFAP and NfL was assessed, while adjusting for sex, BMI Z-score, general cognition, and run batch. Significant interactions were probed with the JN technique to identify the specific age ranges over which EXT was significantly associated with the concentrations of GFAP and NfL.

### Functional brain connectivity

The rsfMRI scan was 6 min long, acquiring 164 time points with a repetition time (TR) of 2.2 s. Of the 137 participants who had usable GFAP/ NfL data, 128 had good quality pre-processed rsfMRI data available (EXT = 42, HC = 86). Participants with high head motion as indexed by mean framewise displacement (FD) > 0.3 mm were excluded. The mean FD did not differ significantly between the EXT and HC groups (EXT: 0.099 ± 0.069; HC: 0.083 ± 0.050; p = 0.156), indicating comparable data quality across groups.

The detailed acquisition protocol and pre-processing pipeline are described elsewhere [29]. Key pre-processing steps included motion correction (FSL MCFLIRT), slice-time correction, brain extraction (FSL BET), ICA-AROMA for denoising, and high-pass filtering (>0.008 Hz). The functional data were co-registered to T1-weighted images, normalized to Montreal Neurological Institute (MNI) space using Advanced Normalization Tools (ANTs), resampled to 2 mm isotropic resolution, and smoothed with a 4 mm Full Width at Half Maximum (FWHM) filter.

Brain connectivity matrices were generated using Analysis of Functional Neuroimages’ (AFNI’s) 3dNetCorr and involved 136 unique between-network connectivity pairs derived from the 17 canonical Yeo-17 functional networks: Visual A/B, Somatomotor A/B, Dorsal Attention A/B, Salience/Ventral Attention A/B, Limbic A/B, Control A/B/C, Default A/B/C, and Temporoparietal [30]. Between-network connectivity was calculated for each participant using full correlation, followed by a Fisher r-to-z transformation on the resulting correlation matrices. Within-network connectivity was not included in the model.

### Normative modeling

Functional normative models were applied on between-network connectivity using the Predictive Clinical Neuroscience toolkit (PCNtoolkit), an open-source python package for normative modelling [31], to generate subject-level deviation scores. These models were originally trained on a multi-site dataset of approximately 22,000 individuals (age range 5-100, 45 sites) using Bayesian Linear Regression, with age, sex, site, and mean FD as covariates. The pre-trained models were first adapted using data from the cVEDA cohort (n = 903; 47% female; age range 5.5–24.3 years; recruited from six sites across India), thereby enabling valid transfer to the present dataset. By transferring these adapted models to the dataset in the current study, deviation (Z) scores for each connectivity feature, representing how much an individual’s connectivity deviates from the expected pattern for their age and sex were generated. These covariates were consistent across all analyses, including the normative modelling pipeline and the moderation/ GLM models, and comprised age, sex, site, and mean FD.

### Multivariate modeling of brain–GFAP/ NfL associations

To identify a sparse set of brain connectivity features associated with GFAP and NfL concentrations, separate sparse Partial Least Squares (sPLS) regressions were conducted using the ‘spls’ package in R. sPLS was chosen for its ability to handle high-dimensional data and perform simultaneous variable selection and association. All predictors were standardized prior to modeling. For each outcome, a three-step procedure was followed: (1) cross-validation (cv.spls) to determine the optimal sparsity (η) and number of components (K); The term “components” refers to the latent variables extracted from the predictor matrix of brain connectivity deviation scores. These components represent orthogonal dimensions capturing shared variance between the predictors and the GFAP/ NfL outcome. K was optimized via cross-validation using the cv.spls function in R.

(2) model fitting with the optimized parameters; and (3) bootstrapping with 10,000 resamples (ci.spls) to identify statistically significant predictors, retaining only those whose 95% confidence intervals excluded zero.

## Results

### Association of EXT with plasma GFAP/ NfL moderated by age

The study included n = 137 subjects from the cVEDA cohort with n = 90 in HC [Age= 15.96 ± 4.55 years, including n = 64 (71.1%) males] and n = 47 in EXT [Age= 15.63 ± 4.21 years, including n = 40 (85.1%) males] groups.

**Fig. 1:**
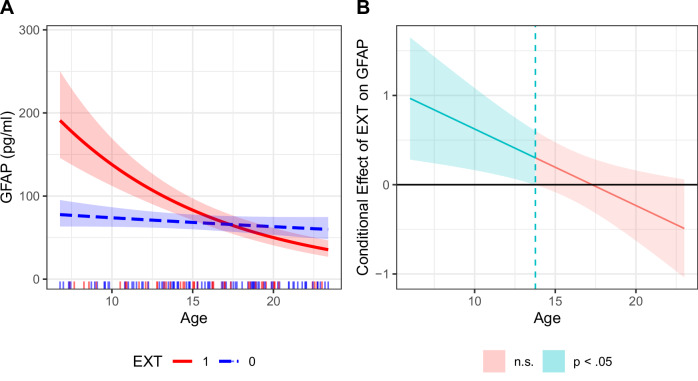
Interaction of Externalizing psychopathology (EXT) and age on Glial fibrillary acidic protein (GFAP) levels. **A** GFAP concentrations (pg/ml) across age, noted for EXT = 1 (red solid line) and EXT = 0 (Healthy controls) (blue dashed line). **B** Conditional effect of EXT on GFAP across the entire age range (Johnson-Neyman plot). Cyan and pink regions denote where the conditional effect of EXT on GFAP is significant (p < 0.05) and non-significant (n.s.), respectively. The vertical dashed line indicates the age threshold for this significance [The number of participants in the externalizing category and $\le$ 14 years (n = 21)], where the 95% confidence interval for the conditional effect of EXT on GFAP includes zero.

The results of the Gamma GLM analyses for GFAP and NfL are presented in Table 2. For GFAP, we observed a significant main effect of EXT, where the EXT group showed higher GFAP concentrations compared to healthy controls [exp(β) = 4.40, 95% CI: 1.83–10.7, p = 0.001]. We also found a significant Age-by-EXT interaction [exp(β) = 0.92, p = 0.002], indicating that the relationship between EXT and GFAP was dependent on age, although the main effect of age alone was insignificant (p = 0.40). Probing this interaction with a JN plot (Fig. 1) revealed that the positive association between EXT and GFAP was significant only in individuals younger than approximately 14 years of age. Additionally, a lower BMI was significantly associated with higher GFAP concentrations across all the samples (n = 137) [exp(β) = 0.88, p = 0.021]. None of the other main effects were significant.

**Table 2:** Gamma GLM analyses for GFAP and NfL.

Parameters	GFAP			NfL		
	exp(Beta)	95% CI	p-value	exp(Beta)	95% CI	p-value
Age (years)	0.98	0.95, 1.02	0.4	1.01	0.96, 1.06	0.7
EXT	4.40	1.83, 10.7	**0.001**	0.97	0.36, 2.73	>0.9
Age (years) x EXT	0.92	0.87, 0.97	**0.002**	1.01	0.95, 1.07	0.8
BMI (Z score)	0.88	0.78, 0.98	**0.021**	0.90	0.79, 1.03	0.093
General Factor Cognition (T-score)	1.00	0.98, 1.02	0.9	1.00	0.98, 1.02	>0.9
Gender (M-F)	0.86	0.66, 1.11	0.3	0.96	0.71, 1.28	0.8
Run (B2-B1)	1.11	0.88, 1.41	0.4	0.98	0.76, 1.27	>0.9

Gamma GLM (log link) results for GFAP and NfL concentrations, indicating Age by EXT interaction, adjusted for sex, BMI Z-score, general factor cognition (gFc_T), and Run. Coefficients are exponentiated (Mean Ratios).

For NfL, there were no significant main effects of age or EXT (Table 2), and the Age-by-EXT interaction was not significant (p > 0.8). None of the covariates were significantly associated with NfL concentrations.

### Association of resting brain networks with plasma GFAP/ NfL

**Fig. 2:**
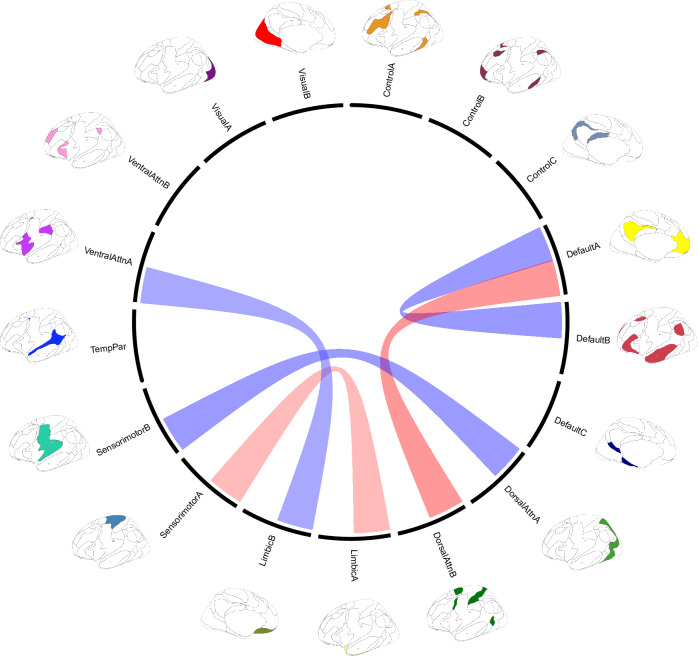
Brain connectivity features associated with plasma GFAP. A Circos plot visualizing the five significant between-network connectivity deviation scores from the sPLS model that was associated with GFAP concentration is shown. The outer ring represents the 17 functional networks from the Yeo atlas. Red links indicate that greater deviation towards stronger connectivity (more positive Z-scores) is associated with higher GFAP. Blue links indicate that greater deviation towards weaker connectivity (more negative Z-scores) is associated with higher GFAP.

The sPLS model examining association of plasma GFAP concentrations with brain connectivity deviation scores identified optimal parameters of η = 0.9, K = 3, where η controls sparsity in variable selection and K is the number of latent components extracted from the 136 between-network connectivity features. The three components (K) explained 19.0%, 4.6%, and 3.1% of the variance in GFAP, respectively, with a total R^2^ of 26.2%. After bootstrap correction, five between-network connectivity features were retained as significantly associated with GFAP (Fig. 2).

Specifically, greater deviation from the normal towards stronger connectivity (i.e., more positive Z-scores) between the Sensorimotor A and Limbic A networks (β = 0.36, 95% CI [0.18, 0.55]) and between the Dorsal Attention B and Default A networks (β = 0.61, 95% CI [0.36, 0.90]) was associated with higher GFAP concentrations. Conversely, greater deviation towards weaker connectivity (i.e., more negative Z-scores) was associated with higher GFAP levels for the connections between the Sensorimotor B and Dorsal Attention A networks (β = −0.33, 95% CI [−0.61, −0.08]), the Ventral Attention A and Limbic B networks (β = −0.21, 95% CI [−0.39, −0.04]), and the Default A and Default B networks (β = −0.36, 95% CI [−0.58, −0.15]).

None of the connectivity features were found to be significantly associated with NfL concentrations.

### GFAP linked connectivity deviations in relation to EXT

Given that case–control comparisons may obscure substantial inter-individual variability in brain organisation in multifactorial conditions such as EXT (Supplementary Figure 1), we focused on an individual level approach. To link connectivity changes to EXT status, we derived a subject level GFAP linked connectivity composite from the sPLS model (Supplementary Methods) and tested its association with EXT in a linear model including age, sex, mean FD, and an age × EXT term. EXT was positively associated with the composite (Estimate = 0.81, 95% CI 0.05–1.57, p = 0.036), and the age × EXT interaction was significant (Estimate = −0.06, 95% CI − 0.10 to −0.01, p = 0.024), indicating that the GFAP related connectivity pattern is elevated in younger EXT participants and converges towards control levels with age (R² = 0.176; Supplementary Figure 2).

## Discussion

In this study, we investigated how externalizing psychopathology (EXT), peripheral indicators of glial activation (GFAP) and axonal injury (NfL), and functional brain connectivity relate to one another. Taken together, our results triangulate an age dependent association between EXT and elevated plasma GFAP with a specific pattern of brain connectivity deviations linked to higher GFAP levels in individuals with EXT, while NfL showed no robust associations with either EXT or connectivity.

A key finding was the significant interaction between age and EXT in relation to GFAP. Specifically, EXT was associated with higher GFAP concentrations in younger participants (under ~14 years of age), with this effect normalizing in older adolescents. This pattern is consistent with a developmental lag hypothesis for externalizing disorders [32–34], compared to the physiological reduction of GFAP which is expected at this age. GFAP levels could be a dynamic biomarker of astrocyte activity, and its elevation in younger individuals with EXT could indicate either delayed astrocyte maturation or a prolonged reactive glial state. That is, astrocytes might be delayed in their shift from glial activation to the normally expected state of glial homeostasis.

The persistence of elevated GFAP up to 14 years could thus point to underlying neurodevelopmental vulnerabilities. One potential mechanism is that immature astrocytes may over-respond to early-life insults (e.g., infections, oxidative stress, adversities), delaying inflammatory resolution and sustaining GFAP upregulation [35, 36]. This, in turn, may disrupt critical astrocyte-dependent processes vital for brain maturation, including synaptic pruning and neural homeostasis [37, 38]. This could parallel the grey matter thinning and atypical white matter maturation documented previously in neurodevelopmental disorders [39, 40]. Given the critical role of glial activation in normal brain sculpting and the established association of altered GFAP with other neurodevelopmental conditions, the observed association between GFAP and EXT in the current study may reflect reverse causality or shared underlying processes between reactive astrogliosis and externalizing disorders. Nevertheless, the specificity of this finding to glial-related processes is reinforced by the absence of similar age- or EXT-related effects on NfL, a marker of neuroaxonal integrity.

Our sPLS analysis identified a multivariate pattern of brain connectivity that associated with GFAP levels, providing insight into the potential neural underpinnings of this glial activation. Higher GFAP was associated with atypically strong connectivity between sensorimotor-limbic and attention-default mode networks (Fig. 2). This pattern suggests a potential dysregulation in the integration of emotion, self-regulation, and motor control circuits, which are central to the manifestation of externalizing behaviours [41, 42]. For instance, the heightened connectivity between limbic and sensorimotor networks could reflect a stronger link between emotional reactivity and action, a hallmark of impulsivity [43].

Conversely, higher GFAP was also associated with weaker-than-expected connectivity (or greater anticorrelation) within the default mode network and between attention and limbic networks. This finding aligns with literature on ADHD, that is characterized by externalizing features like impulsivity and high activity levels, where default mode network (DMN) hypo-connectivity has been linked to behavioural symptoms such as delay aversion [26, 27]. Altered DMN connectivity is often interpreted as a disruption in self-referential thought and introspection [44], and our results suggest this neural signature of dysconnectivity is linked to glial activation in this population.

Given that ADHD and EXT are heterogeneous and multifactorial, it is likely that multiple etiopathogenetic pathways can lead to similar clinical presentations of inattention and disinhibition. Recent work using normative models in ADHD has shown that case control comparisons can obscure substantial inter individual variability in brain biology, with the “average patient” only marginally reflecting the pattern seen in single subjects [45]. Complementary data driven subtyping approaches have also identified distinct neuroimaging subtypes of ADHD with different cognitive profiles, treatment response, and molecular signatures [46], further supporting the presence of multiple biological routes to the same clinical phenotype.

Normative modelling is well suited to this context because it quantifies person specific deviations relative to age and sex adjusted expectations rather than relying solely on group averages. In our data, at group-level the direct EXT–connectivity associations were weak and sparse (Supplementary figure 1), whereas GFAP showed a clear association with EXT and with a specific multivariate pattern of BNFC deviations (Fig. 2). Taken together, these findings indicate that GFAP and its associated multivariate connectivity signature show a coherent relationship with EXT status than individual FC metrics alone in this sample (Supplementary figure 2). We therefore interpret the GFAP linked connectivity pattern as one putative pathway, plausibly related to glial mechanisms, among several possible routes to EXT liability, although the cross-sectional and exploratory nature of these findings should be considered.

Methodologically, our study benefits from the careful matching of EXT and control groups using a Mahalanobis distance-based approach, which controlled for potential confounders including childhood adversity, cognition, age, sex, and BMI. We noted a small but significant negative relationship between GFAP and BMI, yet the primary Age-by-EXT interaction remained significant after accounting for this and other covariates (Table 2). Future research should further explore the interplay among metabolic health, glial function, and the development of externalizing behaviours.

Several limitations of this study warrant consideration. First, the cross-sectional design precludes any inference of causality or temporal direction between GFAP and EXT. Second, the sample size, though adequate for detecting medium-to-large effects, remains modest for high-dimensional multivariate modeling. As highlighted earlier [47], small sample sizes can lead to inflated effect sizes and overly optimistic power estimates. While we employed bootstrapping to enhance robustness, replication in larger, independent cohorts is essential to confirm the reliability and generalizability of our findings. Third, since the sample was predominantly male, the findings may disproportionately reflect male-specific biological patterns and may not be generalised to both the genders. Fourth, GFAP and NfL were treated as exploratory correlates and not validated biomarkers in a clinical sense; future longitudinal studies are needed to assess their utility as risk or monitoring biomarkers. Fifth, the power estimate applies to the main effect and not the interaction term, which requires greater power and external replication. Replication studies in future, in larger, independent cohort would strengthen the robustness of our findings, help address potential inflation of effect sizes, validate the observed associations and to assess the generalizability of the identified connectivity–GFAP patterns. However, due to financial and logistical constraints, we were unable to include an external replication sample in the current study. Furthermore, a significant effect of Age-by-EXT interaction on GFAP, without the main effect of age alone requires further analyses for a conclusive interpretation. While our matching procedure mitigates potential confounding, unmeasured variables could still influence the results. Also, our analysis focused on between-network connectivity, and future studies could explore more fine-grained, region-specific patterns. Lastly, the EXT was primarily composed of individuals with ADHD (53.2%; Table 1), which may skew the findings toward ADHD-related neural and GFAP/ NfL profiles rather than representing EXT more broadly.

In conclusion, our study provides novel evidence linking EXT to age-dependent glial activation. The prolonged GFAP expression in younger individuals with EXT might signify a failure of intrinsic compensatory pathways, perpetuating an atypical neurodevelopmental trajectory. This process is associated with a specific, multivariate signature of brain dysconnectivity, highlighting a potential neurobiological mechanism underlying EXT in youth and suggesting that plasma GFAP may serve as a useful protein indicator of periods of heightened neuro-immune activity in early-stage psychopathology.

## Supplementary information

Supplementary information

## Data Availability

The analysis scripts used in this study are available in a public GitHub repository: https://github.com/hollabharath/cveda-simoa-connectivity.
