# Supplementary material for: Plasma Glial Fibrillary Acidic Protein (GFAP) shows age-dependent associations with externalizing psychopathology and atypical brain connectivity
Source: Transl Psychiatry. 2026 May 29;16:382. doi: 10.1038/s41398-026-04114-2 (PMC13407853; doi:10.1038/s41398-026-04114-2)
Supplement: Supplementary file 1 — Supplementary information [file 41398_2026_4114_MOESM1_ESM.docx]

**Plasma Glial Fibrillary Acidic Protein (GFAP) Shows Age-Dependent Associations with Externalizing Psychopathology and Atypical Brain Connectivity**

**B.S. Niveditha^1,2#^, Bharath Holla^3,#*^, Sarada Subramanian^4^, N. Gagana^2^, K.M Bhargavi^1,2^,** [**Eesha Sharma**](https://pubmed.ncbi.nlm.nih.gov/?sort=date&term=Sharma+E&cauthor_id=31898525)**^5^,  Jayant Mahadevan^6^, Meera Purushottam^7^, Biju Viswanath^6^, Vivek Benegal^6^, Gautham Arunachal^1^, Jon Heron^8^, Matthew Hickman^8^, Debasish Basu^9^, B.N. Subodh^9^, Lenin Singh^10^, Roshan Singh^11^, Kalyanaraman Kumaran^12,13^, Rebecca Kuriyan^14^, Sunita Simon Kurpad^15,16^, Kamakshi Kartik^17^, Kartik Kalyanram^17^, Sylvane Desrivieres^18^, Gareth Barker^19^, Dimitri Papadopoulos Orfanos^20^, Mireille Toledano^21^, Pratima Murthy^6^, Nilakshi Vaidya^22^, Ghattu Krishnaveni^13^, Gunter Schumann^22,23^, Kuldeep Kumar Sharma^24^, BinuKumar Bhaskarapillai^24^, K. Thennarasu^24^, Rajan Kashyap^25^, Rose Dawn Bharath^25^, Amit Chakrabarti^26^, G.K. Chetan^1^, and Muchukunte Mukunda Srinivas Bharath^2,27*^**

^1^ Department of Human Genetics, National Institute of Mental Health and Neurosciences (NIMHANS), No. 2900, Hosur Road, Bengaluru- 560029, India

^2^ Department of Clinical Psychopharmacology and Neurotoxicology, NIMHANS, Bengaluru, India

^3^ Department of Integrative Medicine, NIMHANS, Bengaluru, India

^4^ Department of Neurochemistry, NIMHANS, Bengaluru, India

^5^ Department of Child and Adolescent Psychiatry, NIMHANS, Bengaluru, India

^6^ Department of Psychiatry, NIMHANS, Bengaluru, India

^7^Rohini Nilekani Centre for Brain and Mind, Department of Psychiatry, NIMHANS, Bengaluru, India

^8^Population Health Sciences, Bristol Medical School, University of Bristol, Beacon House, Queens Road, Bristol, BS8 1QU, United Kingdom

^9^ Department of Psychiatry, Post Graduate Institute of Medical Education and Research, Chandigarh-160012, India

^10^ Department of Psychiatry, Regional Institute of Medical Sciences, Lamphel Road, Lamphelpat, Imphal, Manipur-795004, India

^11^ Department of Psychology, Regional Institute of Medical Sciences, Imphal-795004, India

^12^ Primary Care, Population Sciences and Medical Education, University of Southampton, United Kingdom

^13^ Epidemiology Research Unit, CSI Holdsworth Memorial Hospital, Mysuru-570001, India

^14^ Division of Nutrition, St John’s Research Institute, Bengaluru- 560034, India

^15^ Department of Psychiatry, St. John’s Medical College and Hospital, Bengaluru-560034, India

^16^ Department of Medical Ethics, St. John’s Medical College and Hospital, Bengaluru- 560034, India

^17^ Rishi Valley Rural Health Centre, Madanapalle, Chittoor, Andhra Pradesh-517352, India

^18^ Centre for Population Neuroscience and Precision Medicine, Institute of Psychology, Psychiatry & Neuroscience, MRC SGDP Centre, King’s College London, 16 De Crespgny Park, SE5 8AF, London, UK United Kingdom

^19^ Department of Neuroimaging, Institute of Psychology, Psychiatry and Neuroscience, King’s College London, London- SE5 8AF, United Kingdom

^20^ NeuroSpin, CEA, Universit´e Paris-Saclay, Paris, France

^21^ Mohn Centre for Children’s Health and Wellbeing, School of Public Health, Imperial College London, London, United Kingdom.

^22^ Centre for Population Neuroscience and Precision Medicine, Charite Mental Health, Dept. of Psychiatry and Psychotherapy, Charite Universitaetsmedizin Berlin, Germany

^23^ Centre for Population Neuroscience and Precision Medicine, Institute for Science and Technology of Brain-Inspired Intelligence, Fudan University, Shanghai, China

^24^ Department of Biostatistics, NIMHANS, Bengaluru, India

^25^ Department of Neuroimaging and Interventional Radiology, NIMHANS, Bengaluru, India

^26^ ICMR-Centre for Ageing and Mental Health, Indian Council of Medical Research, Block-DP1, Sector-V, Salt Lake, Kolkata - 700 091, India

^27^ Centre for Neurobehavioral Toxicology, Department of Clinical Psychopharmacology and Neurotoxicology, NIMHANS, Bengaluru, India

^#^Contributed equally to the work

*Running title: GFAP and Brain Connectivity in Externalizing Disorders*

*Address for correspondence:

Bharath Holla, MD, PhD, Associate Professor of Psychiatry, Department of Integrative Medicine, NIMHANS, No. 2900, Hosur Road, Bangalore-560029, INDIA; Tel: +91-080-2699-5730; Fax: +91-080-26564830; Email: [hollabharath@gmail.com](mailto:hollabharath@gmail.com); [hollabharath@nimhans.ac.in](mailto:hollabharath@nimhans.ac.in)

M. M. Srinivas Bharath, PhD, Department of Clinical Psychopharmacology and Neurotoxicology, NIMHANS, No. 2900, Hosur Road, Bangalore-560029, INDIA; Tel: +91-080-2699-5113; Fax: +91-080-26564830; Email: [bharath@nimhans.ac.in](mailto:bharath@nimhans.ac.in); [thathachar2010@gmail.com](mailto:thathachar2010@gmail.com)

**Supplementary Methods and Results:**

**Externalizing Psychopathology and Between Network Connectivity in a Case–Control Design**

In this exploratory analysis, for each of the 136 Yeo 17 between network connections, we fitted a linear model with the between network connectivity deviation score (BNFC Z score) as the outcome and Age, EXT status, and their interaction (Age × EXT) as predictors, adjusting for sex and mean framewise displacement (FD). For each model, we obtained regression coefficients with bias corrected and accelerated (BCa) bootstrap 95 % confidence intervals and p values, and computed partial omega squared (ω²) as an effect size for the main and interaction terms. The resulting edge wise estimates were then assembled into a network by network matrix and visualised as a half matrix heatmap to summarise the spatial distribution of EXT related effects.

These analyses revealed several nominal, biologically plausible differences involving control, default, limbic, and sensorimotor networks, but no connection survived correction for multiple comparisons, indicating an absence of a robust, widespread group level shift in BNFC (Supplementary Figure 1). This pattern is consistent with the view that case control contrasts may obscure substantial inter individual variability in brain organisation in multifactorial and heterogeneous conditions such as ADHD and externalizing psychopathology, and highlights the limitations of relying solely on an “average patient” profile.

**Subject level GFAP linked connectivity composite**

To link connectivity changes more directly to EXT status, a subject level GFAP linked connectivity composite was derived, by linearly combining the five BNFC deviation features retained in the GFAP sPLS model using their loadings, such that for each individual,

${GFAP\_connectivity\_composite}_{i}$​=$\sum_{j=1}^{p} w_{j} Z_{ij}$​,

Where,

- **i** indexes individuals,
- **j=1,…,p** indexes the selected connectivity features (here p=5),
- **Z_ij​_** is the BNFC deviation (normative Z score) for feature **j** in individual **i**, and
- **w_j_​** is the corresponding sPLS loading.

The association between this index and EXT in a linear model including age, sex, mean FD, and an age × EXT term was tested as follows: GFAP_connectivity_composite∼EXT×Age+sex+site+mean_FD.

**Supplementary figures**

*
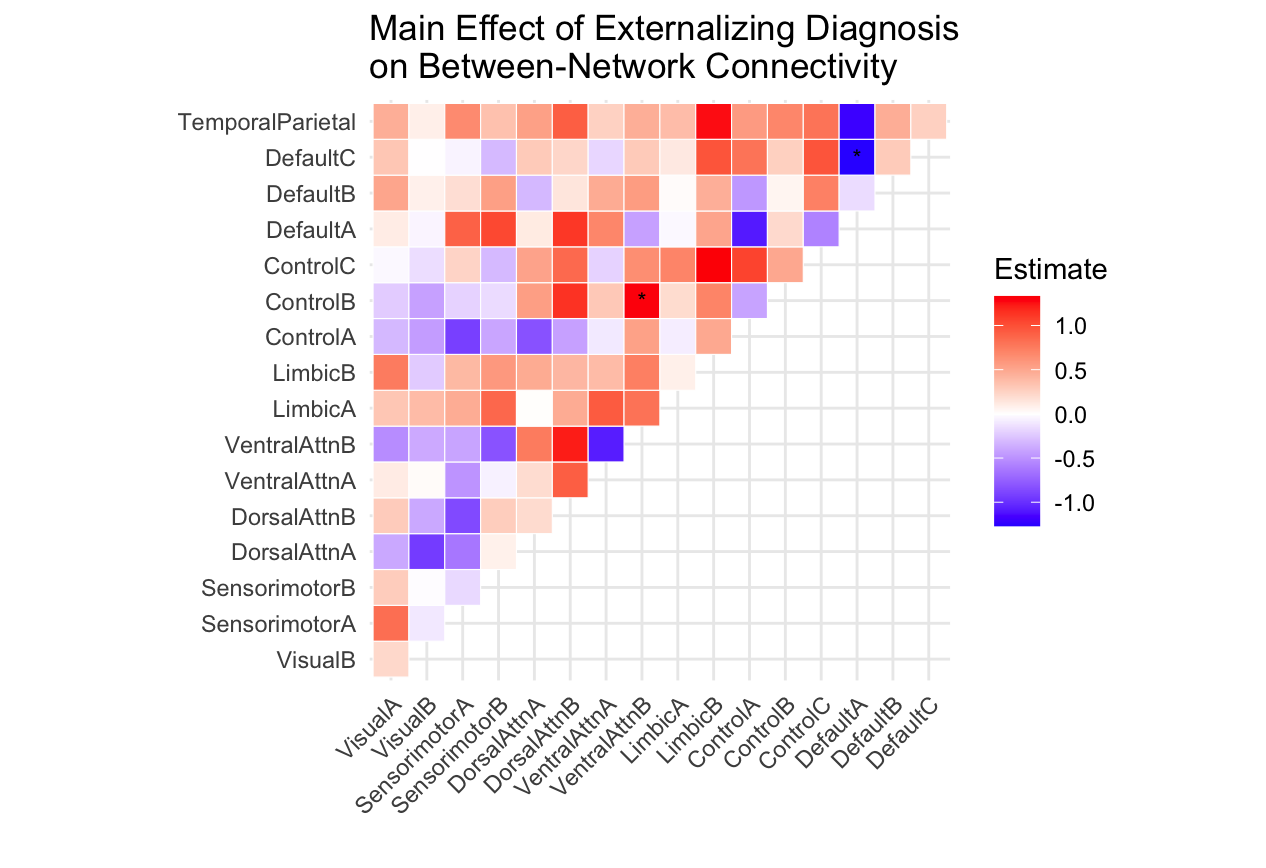
*

**Supplementary Figure 1. Main effect of EXT on between-network connectivity deviation scores.** Heatmap showing regression coefficients for the effect of EXT vs HC on between-network connectivity (BNFC) Z scores derived from the Yeo-17 network normative model. Each cell represents the EXT coefficient from linear models of the form BNFC Z score ~ EXT + age + sex + site + mean FD. Warmer colors indicate more positive deviations in EXT (stronger-than-expected connectivity relative to the normative model), and cooler colors indicate more negative deviations (weaker-than-expected connectivity). Asterisks mark edges with nominal significance at p < 0.05 (uncorrected); no edges survived correction for multiple comparisons.

**

**Supplementary Figure 2**. Age dependent associations between GFAP, connectivity, and externalizing status. (A) Relationship between age and plasma GFAP concentrations (log10 scale) in externalizing (open circles, dashed line) and control (filled circles, solid line) participants. Shaded bands indicate 95% confidence intervals of the group wise linear fits. (B) Association between plasma GFAP concentration (log10 scale) and the GFAP linked connectivity composite derived from the sPLS model, showing a positive linear relationship. (C) Relationship between age and the GFAP linked connectivity composite, again plotted separately for EXT and control (HC) groups (open vs filled circles, dashed vs solid lines). All panels use normative deviation-based connectivity indices and illustrate that both GFAP levels and the associated connectivity pattern are elevated in younger externalizing participants and converge toward control levels with increasing age.
